# Physically aggressive conspiracy believers often faced various psycho-social problems: Findings from a longitudinal population-based study

**DOI:** 10.1371/journal.pone.0353935

**Published:** 2026-09-09

**Authors:** Peter G. van der Velden, Arno van Dam, Jan-Jaap van Eerten, Koen P. Grootens

**Affiliations:** 1 Tranzo, Tilburg School of Social and Behavioral Sciences, Tilburg University, Tilburg, The Netherlands; 2 Centerdata, Tilburg, The Netherlands; 3 Dutch National Support Center for Extremism (LSE), Zeist, The Netherlands; 4 GGZ WNB Mental Health Institute, Halsteren, The Netherlands; 5 Reinier van Arkel Mental Health Institute, ‘s Hertogenbosch, The Netherlands; Yeditepe University, TÜRKIYE

## Abstract

**Objectives:**

Psychosocial problems are positively associated with beliefs in conspiracy theories and physically aggressive behavior that are mutually related. Yet, it is unknown if adults with (a higher number of) these problems are more often aggressive conspiracy believers, non-physically aggressive conspiracy believers and aggressive non-believers than non-physically aggressive non-believers. The present study aims to fill this gap of knowledge.

**Methods:**

Data was extracted from surveys conducted with the Dutch population-based LISS panel assessing six pre-existing psychosocial problems (severe anxiety and depression symptoms, lack of support, exposure to traumatic and stressful life events, financial problems, and low levels of life satisfaction). Furthermore, data was extracted from a survey 5–7 months later assessing conspiracy beliefs and physical aggression. Based on the data of the last survey we distinguished non-physically aggressive non-conspiracy believers, non-physically aggressive believers, physically aggressive non-believers and physically aggressive believers (N^total^ = 1,385). Series of multivariate multinomial logistic regression analyses were conducted with group-membership as dependent variable and (the number of) psychosocial problems as predictors.

**Results:**

Results showed that respondents with pre-existing psychosocial problems were much more often aggressive non-conspiracy believers and aggressive conspiracy believers than non-physically aggressive non-believers. Analyses among the physically aggressive believers and non-physically aggressive non-believers revealed that about 64% of the respondents with four or more problems were physically aggressive believers compared to 4% without these problems. The assessed (number of) psychosocial problems did not distinguish non-physically aggressive non-believers and non-physically aggressive believers. Using different cut-offs scores to identify the four groups hardly changed found patterns.

**Conclusions:**

Findings indicate that the higher the number of pre-existing psychosocial problems the more adults are prone to be physically aggressive conspiracy believers and, to a lesser extent, physically aggressive non-believers. Results suggest that policies and interventions to reduce violence and the adoption and dispersion of conspiracy beliefs should in any case, besides other potential measures, be aimed at preventing and reducing pre-existing psychosocial problems.

## Introduction

Conspiracy theories proclaim that secret influential organizations or powerful groups are responsible for important specific often malevolent events or phenomenon’s [1,2]. “*It is a subset of false beliefs in which the ultimate cause of an event is believed to be due to a plot by multiple actors working together with a clear goal in mind, often unlawfully and in secret*” [3].

Research has shown that beliefs in conspiracy theories are associated with antisocial behavior, varying from willingness to file false insurance claims and reduced adherence to public health regulations to illegitimate forms of political engagement such as intentions to violently protest, physically attack police officers, and harass other-minded people online [4–9]. The antisocial (aggressive) behavior is generally specifically directed against the subject or persons to whom the conspiracy theory applies [10]. In addition, beliefs in conspiracy theories are also associated with reduced prosocial behavior such as donations to charity and volunteerism [11]. As a result, serious concerns are increasing worldwide about the adoption and dispersion of conspiracy theories and possible negative effects of these beliefs [11,12]. It is estimated that between 10% (Iceland) and 55% (Bulgaria) of the residents of European countries, aged 15 years and older, incorporated such theories to a greater or lesser extent [2].

A recent review on the relationship between belief in conspiracy theories and violence shows a weak association between belief in conspiracy theories and violent attitude [13]. Moreover, in these studies the conceptualizations of violence used differed significant and comprised mostly attitudes instead of behavior like intentions to commit violence [13]. Measurement of actual violent behavior is scarce and has thus far been confined to extremist offender data and has not been studied in community samples. This means that although there is an association between conspiracy thinking and aggression, many individuals who belief in conspiracy beliefs will not resort to aggression. Rather than looking for weak correlations between these variables, it is more useful to investigate which conspiracy believers actually engage in aggressive behavior and which circumstances are associated with the coexistence of these phenomena. For organizations focusing on interventions aimed at preventing violence, it is also more relevant to look within the population at individuals who exhibit highly aggressive behavior and strongly believe in conspiracy theories than at weak associations between variables in individuals who do not pose a risk to safety.

Insight in (modifiable) factors that are associated with or contribute to individuals’ beliefs in conspiracy theories is warranted to be able to develop and target policies and interventions to reduce the adoption and dispersion of conspiracy beliefs and their negative effects. Studies showed that unfavorable life conditions such as mental health problems, (adverse) childhood experiences, stressful life events, lack of self-esteem, weaker social networks, low SES/financial situation, emotional neglect, are related to both beliefs in conspiracy theories and anti-social or aggressive behavior [14–23]. These findings are in line with the 3N (Needs, Narratives, Network) framework of Kruglanski and colleagues, positing that belief in conspiracy theories has a motivational function. According to the 3N model, narratives, including conspiracy theories, can satisfy a person's needs and provide a social network that approves of the person. For individuals with unfavorable life circumstances, conspiracy theories may satisfy their need to maintain a positive self-image. The use of violence may satisfy the need for significance through aggressive behavior against people who are a threat to their self-esteem [24].

These unfavorable life conditions (hereafter abbreviated as psychosocial problems) may be the common factor in the positive relationships between beliefs in conspiracy theories on the one hand and (physical) aggressive behavior on the other. In addition, conspiracy theories may provide a rationale and outlet for hostile and aggressive individuals. Moreover, belief in conspiracy theories may be a relevant factor in pathways toward radicalization [25–27].

Aim of the present longitudinal population-based study was to further unravel the relationships between pre-existing psycho-social problems on the one hand, and beliefs in conspiracy theories and physically aggressive behavior on the other hand. For this purpose, unlike other studies, we differentiated between:

1.) non-physically aggressive non-conspiracy believers (subgroup 1),
2.) non-physically aggressive conspiracy believers (subgroup 2),
3.) physically aggressive non-conspiracy believers (subgroup 3), and
4.) physically aggressive conspiracy believers (subgroup 4).

By distinguishing these four subgroups, the extent to which pre-existing psycho-social problems increase risk of being a conspiracy believer, of being physically aggressive and/or of being a physically aggressive conspiracy believer can more accurately be determined than by entering both variables in the analyses separate and simultaneously.

In line with aforementioned research, we focused on the following six pre-existing psycho-social problems and the number of these problems as predictors: anxiety and depression symptomatology, lack of emotional support, potential traumatic events (in the past 12 months), stressful life events (in the past 12 months), financial problems, and life satisfaction.

### Hypothesis

We tested the following five complementary hypothesis. Respondents with pre-existing problems and a higher number of pre-existing problems

- H1: are more often non-physically aggressive conspiracy believers (subgroup 2) than non-physically aggressive non-conspiracy believers (subgroup 1),
- H2: are more often physically aggressive non-conspiracy believers (subgroup 3) than non-physically aggressive non-conspiracy believers (subgroup 1),
- H3 are more often physically aggressive conspiracy believers (subgroup 4) than non-physically aggressive non-conspiracy believers (subgroup 1),
- H4: are less often physically aggressive non-conspiracy believers (group 3) than physically aggressive conspiracy believers (subgroup 4), and
- H5: are less often non-physically aggressive conspiracy believers (subgroup 2) than physically aggressive conspiracy believers (subgroup 4).

## Methods

### Procedures and participants

For the present study data was extracted from three surveys conducted with the Dutch *Longitudinal Internet studies for the Social Sciences* (LISS) panel that started in 2007 [28]. This panel is based on a large traditional probability sample drawn from the Dutch population register by Statistics Netherlands (CBS). Individuals and households who do not have Internet access are provided with a broadband connection and a computer called the “simPC”. The simPC is a small and simple device that makes use of centralized support and maintenance.

More specifically, we extracted data from the 2023 survey of the longitudinal VICTIMS-study [29] conducted in March-April (T1a, N^invited^ = 6,409, response = 82.3%, accessed 8/6/2023), from the 2023 survey of the yearly Core Personality study conducted in May-June 2023 (T1b, N^invited^ = 6,518, response = 79.2%, accessed 26/7/2023) and from a survey on conspiracy beliefs and physical aggression conducted in October-November 2023 (T2, N^invited^ = 6,128, response = 83.8%, accessed 1/12/2023). Of the adults 5,272 respondents at T1a, 4,388 participated (with complete data) in the two other surveys (response = 83.2%). The surveys of these studies were administered at the first Monday of the first month.

Respondents receive an incentive of €15 per hour for their participation. For further information about the open-access data of surveys conducted with the LISS-panel see the LISS data archive at https://www.dataarchive.lissdata.nl/ (in English, see Data availability statement). With respect to data security, Centerdata is ISO 27001 and NEN 7510 certified, and the LISS data archive has obtained the CoreTrustSeal certification.

To optimize the representativeness of the study sample, data were weighted for the Dutch adult population using 56 exclusive demographic profiles of the total Dutch adult population [29] on the distribution of sex (males and females), age (14 age categories), and marital status (married, unmarried; January 2023). The applied weights are based on the open access data of Statistics Netherlands (CBS), on gender, age (18 years or older; see https://opendata.cbs.nl/statline/#/CBS/en/).

All results are based on the weighted sample.

### Ethical approval and informed consent

The study on conspiracy beliefs and physical aggression was approved by the Ethical Review Board of the Tilburg School of Social and Behavioral Sciences, The Netherlands (TSB-RP1144). The surveys of the longitudinal VICTIMS-study (that started in 2018) was approved by an Internal Review Board of Centerdata consisting of independent, internal and external reviewers. These reviewers were not involved in the development of the VICTIMS-study. With respect to the longitudinal Core study Personality (this study started at the beginning of the LISS panel in 2008), the survey was approved by the Board of Overseers, an Internal Review Board (IRB) of the LISS panel until 2014.

In accordance with the General Data Protection Regulation (GDPR), participants gave explicit digital consent for the use of the collected data for scientific and policy relevant research (participants of the original probability sample or refreshment samples after 2007). Only when respondents agree to the LISS informed consent they can become a LISS panel member, and only panel members are administered the surveys (see https://www.lissdata.nl/ethics). For the open access data of Statistics Netherlands (CBS) related to the demographics of the total Dutch population, informed consent is not applicable.

The authors assert that all procedures contributing to this work comply with the ethical standards of the relevant national and institutional committees on human experimentation and with the Helsinki Declaration of 1975, as revised in 2008.

### Measures

**Demographics.** Data on sex, age, marital status, primary occupation, and education level at T1a, were used in the present study.

**Physically aggressive behavior.** Physically aggressive behavior in the past 12 months was examined at T2 using the 10-item subscale Physically aggression of the Subtypes of Antisocial Behavior Questionnaire [30] was administered at T2 to examine physically aggressive behavior. Examples of items are “*Hit back when hit by others*”, “*Got into fights more than the average person*” and “*Got into physical fights*” and “*Threatened others*”. Items have 5-point Likert scales (0 = never, 1 = hardly ever, 2 = sometimes, 3 = often, 4 = very often; Cronbach’s alpha = 0.809).

**Conspiracy beliefs.** Conspiracy beliefs at T2 were examined using the 15-item Generic Conspiracist Beliefs Scale [31,32]. For the present study we focused on the 12 non-extraterrestrial items, such as “*The spread of certain viruses and/or diseases is the result of the deliberate, concealed efforts of some organization*”. Items have 5 point Likert scales (1 = definitely not true, 2 = probably not true, 3 = I do not know, 4 = probably true, 5 = definitely true; Cronbach’s alpha = 0.947).

**Anxiety and depression symptoms.** Anxiety and depression symptoms at T1a were using the 5-item Mental Health Inventory [33]. Respondents rated their mental health during the past month on 6-point Likert scales (0 = never to 5 = permanently; Cronbach’s alpha = 0.879). After recoding the three negatively formulated items, the total scores were computed and multiplied by four (to arrive at a 0–100 scale). Scores between 0 and 44 indicate severe symptoms levels, between 45 and 60 as moderate symptom levels, and between 61 and 100 as no-very low symptom levels [34].

**Lack of emotional support.** Lack of emotional support at T1b from people respondents interact with (such as family members, friends, acquaintances, neighbors, and colleagues) was examined using the 8-item subscale Lack of emotional support of the Social Support List-Discrepancy [35,36]. The SSL-D items apply 4-point Likert scales (1 = I miss it, I would like it to happen more often to 4 = It happens too often, it would be nice if it happened less often; Cronbach’s Alpha = 0.888). For the present study, total scores were subtracted from the total maximum scores whereby higher scores reflect more lack of emotional support. These scores were recoded into no-low lack (scores 0 thru 9), medium lack (scores 10 thru 13), and high lack of emotional support (14 thru hi) [37].

**Traumatic and stressful life events.** Potential traumatic (PTE) and stressful life events in the 12 months before T1a were examined at T1a by a list of 21 events (1 = yes, 2 = no) based on pre-existing research on PTEs [29]. Respondents could add an event not listed and this event was recoded in new or existing type of event. PTEs in the past 12 months included the following events (meet criterion A of DSM-5): serious threats, (sexual) violence/abuse, and (traffic) accidents. A distinction was made between respondents who were exposed to a PTE (one or more) in the past 12 months or not (1 = no, 2 = yes). Stressful life events in the past 12 months included (online) theft, burglary, unexpected death loved one or colleague, expected death loved one or colleague, contraction of a serious infection (e.g., HIV, AIDS), development of a serious physical ailment (e.g., cancer, heart attack), and other events not listed (such as divorce, loss of job, serious mental health problems loved one). A distinction was made between respondents who were exposed to a stressful life event (one or more) in the past 12 months or not (1 = no, 2 = yes).

**Financial problems.** Financial problems were examined at T1a with the brief Problems and Help Inventarisation List [29]. The PHIL examines physical problems, mental health problems, problems at work, religious problems, problems with partner/family, legal problems, administrative problems, and financial problems (1 = yes, 2 = no) with the following instruction “People can experience different types of problems. Please indicate for each of the problem types listed below whether you experience these problems or not”.

**Life satisfaction.** Life satisfaction at T1b was examined with the 5-item Satisfaction with life scale [38]. Items have 7-point Likert scales (1 = strongly disagree to 7 = strongly agree; Cronbach’s alpha = 0.902). For the present study sum scores were divided in five exclusive descending categories: very high (30 thru hi = 1), high (28 thru 29 = 2), medium (25 thru 27 = 3), low (21 thru 24 = 4), and very low scores (5 thru 20 = 5), each covering about 20% of life satisfaction scores.

### Data analyses

In the present study group membership (non-physically aggressive non-conspiracy believers, physically aggressive non-conspiracy believers, non-physically aggressive conspiracy believers, physically aggressive conspiracy believers) is the dependent variable. However, to the best of our knowledge there are no validated cut-off scores available to distinguish respondents with and without conspiracy beliefs and distinguish respondents who are physically aggressive or not, to be able to distinguish there four subgroups. Because we focused on these specific four groups, Latent Profile Analysis (LPA) was not applied because LPA is aimed at determining if and which unobserved clusters of respondents (latent classes) exists who are similar in how they respond to a set of continuous input variables (indicators, in this case conspiracy beliefs and physical aggression scores), which was not the aim of the present study.

To be able to identify these four groups and simultaneously prevent as much as possible that results are predominantly determined by a chosen cut-off score, we used three consecutive cut-off beliefs scores to identify conspiracy believers and three consecutive cut-off aggression scores to identify those who are physically aggressive in the statistical analyses. Respondents who scored zero on the scales were considered non-believers and non-physically aggressive respondents.

Because the Generic Conspiracist Beliefs Scale and Antisocial Behavior Questionnaire have different Likert scales, we first dichotomized each item of the conspiracy beliefs questionnaire into “does not have the specific belief” (0 = 1, 2 or 3) and “does have the specific belief” (1 = 4 or 5). We next calculated the total score of the 15 items. Higher scores indicate more conspiracy beliefs. As said, respondents were considered to be non-conspiracy believers when their sum score was zero (did not have any assessed conspiracy belief). We applied the following three cut-off sum scores: ≥ 6 reflecting 13% highest scores, ≥ 5 reflecting 18% highest scores, and ≥ 4 reflecting 25% highest scores (for readability hereafter abbreviated as conspiracy believers). In a similar way, respondents were considered not being physically aggressive when their aggression sum score was zero (all aggression items were answered with “never”). To identify physically aggressive respondents (higher scores indicate more physically aggressive behavior), a cut-off sum score of ≥ 6 reflecting 13% highest scores, a sum score of ≥ 5 reflecting 17% highest scores, and a sum of score ≥ 4, reflecting 22% highest scores was applied.

To test the hypotheses, multivariate multinomial logistic regression (MMLR) analyses were conducted with group membership as dichotomous dependent variables (subgroup 1 – subgroup 2; subgroup 1 – subgroup 3; subgroup 1- subgroup 4; and subgroup 4 – subgroup 3; subgroup 4 – subgroup 2) and pre-existing problems at T1a as predictors. Sex, education level, marital status, employment status, and age in years at T1a were treated as control variables totaling 6 predictors in each MMLR. For each pre-existing problem a separate MMLR analyses was conducted to limit the number of predictors in the analyses (events-per-variable ratio). We furthermore examined the extent to which a higher number of assessed pre-existing problems (range 0–4 or more) is associated with being a (physically aggressive) conspiracy believer. In the first series of MMLR analysis, the adjusted Odd Ratios (aORs) were computed relative to group 1 (non-physically aggressive non-conspiracy believers) totaling seven MMLR analyses (H1, H2, H3). The analyses were repeated for H4 and H5 in which the aORs were computed relative to group 4 (physically aggressive conspiracy believers). Because of the high number of comparison (p-values, n = 75) we applied the Benjamini-Hochberg correction of p-values [39].

We started the MMLR using the highest cut-off scores (≥ 6). Based on the dichotomizations using this cut-off score for both beliefs and physically aggressive behavior, the following four exclusive groups: 1.) non-physically aggressive non-conspiracy believers (group 1, N = 894), 2.) non-physically aggressive conspiracy believers (group 2, N = 139), 3.) physically aggressive non-conspiracy believers (group 3, N = 197), and 4.) physically aggressive conspiracy believers (group 4, N = 129). We repeated the MMLR analyses using the described lower cut-off scores. The analyses using the lower cut-off scores were used to test the robustness of the results using the highest cut-off scores (see section Robustness of findings).

The analyses were performed with IBM SPSS version 28.

## Results

### Characteristics distinguished groups

The demographic characteristics of the four distinguished groups using the highest cut-off score (≥ 6) for both beliefs and physically aggressive behavior are presented in Table 1, showing that the four groups significantly differed in the distribution of age-categories, being married or not, being employed or not, and low, medium and high education levels.

**Table 1:** Demographic characteristics of four subgroups at T1a.

	Group 1: *Non-physically aggressive non-conspiracy believers* (N = 894)	Group 2: *Non-physically aggressive conspiracy believers* (N = 139)	Group 3: *Physically aggressive non-conspiracy believers* (N = 197)	Group 4: *Physically aggressive conspiracy believers* (n = 129)
	n (%)	n (%)	n (%)	n (%)
Sex				
- male	422 (47.2)	68 (48.9)	107 (54.6)	65 (50.8)
- female	472 (52.8)	71 (51.1)	89 (45.4)	63 (49.2)
Age				
− 18–34 years	218 (24.4)	21 (15.1)	82 (41.6)	51 (39.5)^***^
− 35–49 years	161 (18.0)	29 (20.9)	68 (34.5)	43 (33.3)
− 50–64 years	262 (29.3)	40 (28.8)	32 (16.2)	23 (17.8)
− 65 years and older	253 (28.3)	49 (35.3)	15 (7.6)	12 (9.3)
Marital status				
- married	401 (44.9)	64 (46.0)	75 (38.1)	44 (34.1)^*^
- unmarried	493 (55.1)	75 (54.0)	122 (61.9)	85 (65.9)
Primary occupation				
- employed	483 (54.0)	57 (41.0)	118 (60.2)	84 (65.1)^***^
- not employed	411 (46.0)	82 (59.0)	78 (39.8)	45 (34.9)
Education level				
- low	211 (23.6)	54 (38.8)	52 (26.4)	29 (22.7)^***^
- medium	311 (34.7)	46 (33.1)	87 (44.2)	57 (44.5)
- high	373 (41.7)	39 (28.1)	58 (29.4)	42 (32.8)

* *p* < 0.05, *** *p* < 0.001.

Due to the weighting, numbers may differ slightly.

### Pre-existing psychosocial problems and group membership

The results of the MMLR analyses with respect to H1 to H3 are presented in Table 2 (for the 95% confidence intervals of the adjusted Odd ratios and p-values we refer to Appendix 1).

**Table 2:** Results multivariate multinomial logistic regression analysis Part 1.

			Adjusted OR (95% CI) relative to the Group 1 *Non-physically aggressive non-conspiracy believers*					
		Group 1 (n = 894)	Group 2: *Non-physically aggressive conspiracy believers* (n = 139)		Group 3: *Physically aggressive non-conspiracy believers* (n = 197)		Group 4: *Physically aggressive conspiracy believers* (n = 129)	
	N^total^	n	n (%)	AOR	n (%)	AOR	n (%)	AOR
Anxiety and depression symptoms levels (T1a)								
- no (ref.)	1,014	741	122 (14.1)	1	96 (11.5)	1	55 (6.9)	1
- moderate	242	125	14 (10.1)	0.64	67 (34.9)	3.65^***^	36 (22.4)	3.66^***^
- high	102	28	3 (9.7)	0.49	34 (54.8)	9.40^***^	37 (56.9)	19.81^***^
Lack of emotional support (T1a)								
- no (ref.)	889	649	97 (13.0)	1	99 (13.2)	1	44 (6.3)	1
- medium	260	154	26 (14.4)	1.12	49 (24.1)	2.03^***^	31 (16.8)	2.92^***^
- high	209	90	16 (15.1)	1.11	49 (35.3)	3.66^***^	54 (37.5)	8.95^***^
Potential traumatic events (in 12 months before T1a)								
- no (ref.)	1,245	855	133 (13.5)	1	168 (16.4)	1	89 (9.4)	1
- yes	112	39	5 (11.4)	0.90	29 (42.6)	3.55^***^	39 (50.0)	9.25^***^
Stressful life events (in 12 months before T1a)								
- no (ref.)	978	669	103 (13.3)	1	130 (16.3)	1	76 (10.2)	1
- yes	378	224	35 (13.5)	1.01	66 (22.8)	2.37^***^	53 (19.1)	3.10^***^
Financial problems (T1a)								
- no (ref.)	1,280	873	134 (13.3)	1	176 (16.8)	1	97 (10.0)	1
- yes	79	21	5 (19.2)	1.16	21 (50.0)	5.21^***^	32 (60.4)	16.13^***^
Life satisfaction (T1b)								
- very high (ref.)	375	298	38 (11.3)	1	26 (8.0)	1	13 (4.2)	1
- high	201	159	17 (9.7)	0.91	18 (10.2)	1.16	7 (4.2)	0.98
- medium	279	179	27 (13.1)	1.10	49 (21.5)	2.79^***^	24 (11.8)	3.00^**^
- low	226	125	27 (17.8)	1.74^*1^	48 (27.7)	4.01^***^	26 (17.2)	4.45^***^
- very low	276	133	29 (17.9)	1.54	56 (29.6)	4.74^***^	58 (30.4)	10.45^***^
Number of assessed pre-existing psychosocial problems								
- zero (ref.)	438	352	42 (10.7)	1	30 (7.9)	1	14 (3.8)	1
- one	395	276	56 (16.9)	1.62^*^	48 (14.8)	2.39^***^	15 (5.2)	1.64
- two	265	170	22 (11.5)	1.03	44 (20.6)	3.47^***^	29 (14.6)	4.78^***^
- three	138	68	14 (17.1)	1.61	34 (33.3)	7.35^***^	22 (24.4)	10.13^***^
- four or more	121	28	4 (12.5)	1.20	40 (58.8)	20.28^***^	49 (63.6)	58.10^***^

ref. = reference category. T1a = March-April 2023. T1b = May-June 2023. T2 = October-November 2023. aOR = Odds Ratio adjusted for sex, education level, marital status, employment status, and age at T1a. Data is weighted for the Dutch adult population (18+), based on 56 demographic profiles (sex, marital status, age). Due to the weighting, numbers may differ slightly.

^1^ Not significant after the Benjamini-Hochberg correction of p-values.

* *p* < 0.05, ** *p* < 0.01, *** *p* < 0.001.

In Table 2 in column N^total^, the total number of respondents with different levels of pre-existing problems are presented. In column “Group 1”, next to the number of respondents of group 1 (n) of each level of pre-existing problems, three percentages are presented (%/% /%). The first percentages relate to the comparison of group 1 and 2, the second to group 1 and, and the third to group 1 and group 4 (and thus totaling 100% with the percentages of group 2, 3, and 4 respectively in the same row).

The outcomes show that among the non-physically aggressive respondents (group 1 and group 2), those with pre-existing problems were not more often conspiracy believers (group 2) than those without these pre-existing problems. For instance, of the non-physically aggressive respondents who were and were not exposed to stressful life events a similar percentage (13.3% and 13.5%) were conspiracy believers (aOR = 1.01, p = 0.97). The results with respect to low satisfaction and having two pre-existing problems were no longer significant after the Benjamini-Hochberg correction.

Respondents with these pre-existing problems were, regardless of the nature of the problem, more likely to be physically aggressive non-conspiracy believers (group 3) and physically aggressive conspiracy believers (group 4) than those without these pre-existing problems. With respect to potential traumatic events for example, victims of these events were more like to be physically aggressive non-conspiracy believers (44.6% versus 16.4%) and physically aggressive conspiracy believers (51.4% versus 9.7%) than respondents who were not victimized by these events in the 12 months before T1a. Moreover, compared to respondents without any of the assessed pre-existing problems, those with four or more pre-existing problems more often were physically aggressive non-conspiracy believers (58.8% versus 7.9%) and physically aggressive conspiracy believers (63.6% versus 3.8%) than non-physically aggressive non-conspiracy believers.

In Table 3 the results are presented of the MMLR analyses with respect to H4 and H5 in a similar way as in Table 2 (for the 95% confidence intervals of the adjusted Odd ratios and p-values we refer to S1 and S2 Appendix). Findings show that among physically aggressive respondents (group 3 and group 4) those with severe anxiety and depression symptoms, with high lack of emotional support, being victimized by potentially traumatic events, having financial problems, and having four or more pre-existing problems, were significantly less often non-conspiracy believers.

**Table 3:** Results multivariate multinomial logistic regression analysis Part 2.

			Adjusted OR (95% CI) relative to Group 4 *Physically aggressive conspiracy believers*			
		Group 4 (n = 129)	Group 3:*Physically aggressive non-conspiracy believers* (n = 197)		Group 2:*Non-physically aggressive conspiracy believers* (n = 139)	
	N^total^	n	n (%)	AOR	n (%)	AOR
Anxiety and depression symptoms levels (T1a)						
- no (ref.)	1014	55	96 (63.6)	1	122 (68.9)	1
- moderate	242	36	67 (65.0)	1.00	14 (28.0)	0.17^***^
- high	102	37	34 (47.9)	0.47^*^	3 (7.5)	0.02^***^
Lack of emotional support (T1a)						
- no (ref.)	889	44	99 (69.2)	1	97 (68.8)	1
- medium	260	31	49 (61.3)	0.70	26 (45.6)	0.38^**^
- high	209	54	49 (47.6)	0.41^***^	16 (22.9)	0.12^***^
Potential traumatic events (in 12 months before T1a)						
- no (ref.)	1245	89	168 (65.4)	1	133 (59.9)	1
- yes	112	39	29 (42.6)	0.38^**^	5 (11.4)	0.10^***^
Stressful life events (in 12 months before T1a)						
- no (ref.)	978	76	130 (63.1)	1	103 (57.5)	1
- yes	378	53	66 (55.5)	0.76	35 (39.8)	0.33^***^
Financial problems (T1a)						
- no (ref.)	1280	97	176 (64.5)	1	134 (58.0)	1
- yes	79	32	21 (39.6)	0.32^***^	5 (13.5)	0.07^***^
Life satisfaction (T1b)						
- very high (ref.)	375	13	26 (66.7)	1	38 (74.5)	1
- high	201	7	18 (72.0)	1.18	17 (70.8)	0.93
- medium	279	24	49 (67.1)	0.93	27 (52.9)	0.37^*^
- low	226	26	48 (64.9)	0.90	27 (50.9)	0.39^*^
- very low	276	58	56 (49.1)	0.45^*1^	29 (33.3)	0.15^***^
Number assessed pre-existing problems psychosocial problems						
- zero (ref.)	438	14	30 (68.2)	1	42 (75.0)	1
- one	395	15	48 (76.2)	1.45	56 (78.9)	0.98
- two	265	29	44 (60.3)	0.73	22 (43.1)	0.21^***^
- three	138	22	34 (60.7)	0.73	14 (38.9)	0.16^***^
- four or more	121	49	40 (44.9)	0.35^**^	4 (7.5)	0.02^***^

Ref. = reference category. T1a = March-April 2023. T1b = May-June 2023. T2 = October-November 2023. aOR = Odds Ratio adjusted for sex, education level, marital status, employment status, and age at T1a. Data is weighted for the Dutch adult population (18+), based on 56 demographic profiles (sex, marital status, age). Due to the weighting numbers may differ slightly. Difference between group 4 and group 1 not shown in Table 3, for differences between these groups see Table 2.

^1^ Not significant after the Benjamini-Hochberg correction of p-values.

* *p* < 0.05. ** *p* < 0.01. *** *p* < 0.001.

**Fig 1:**
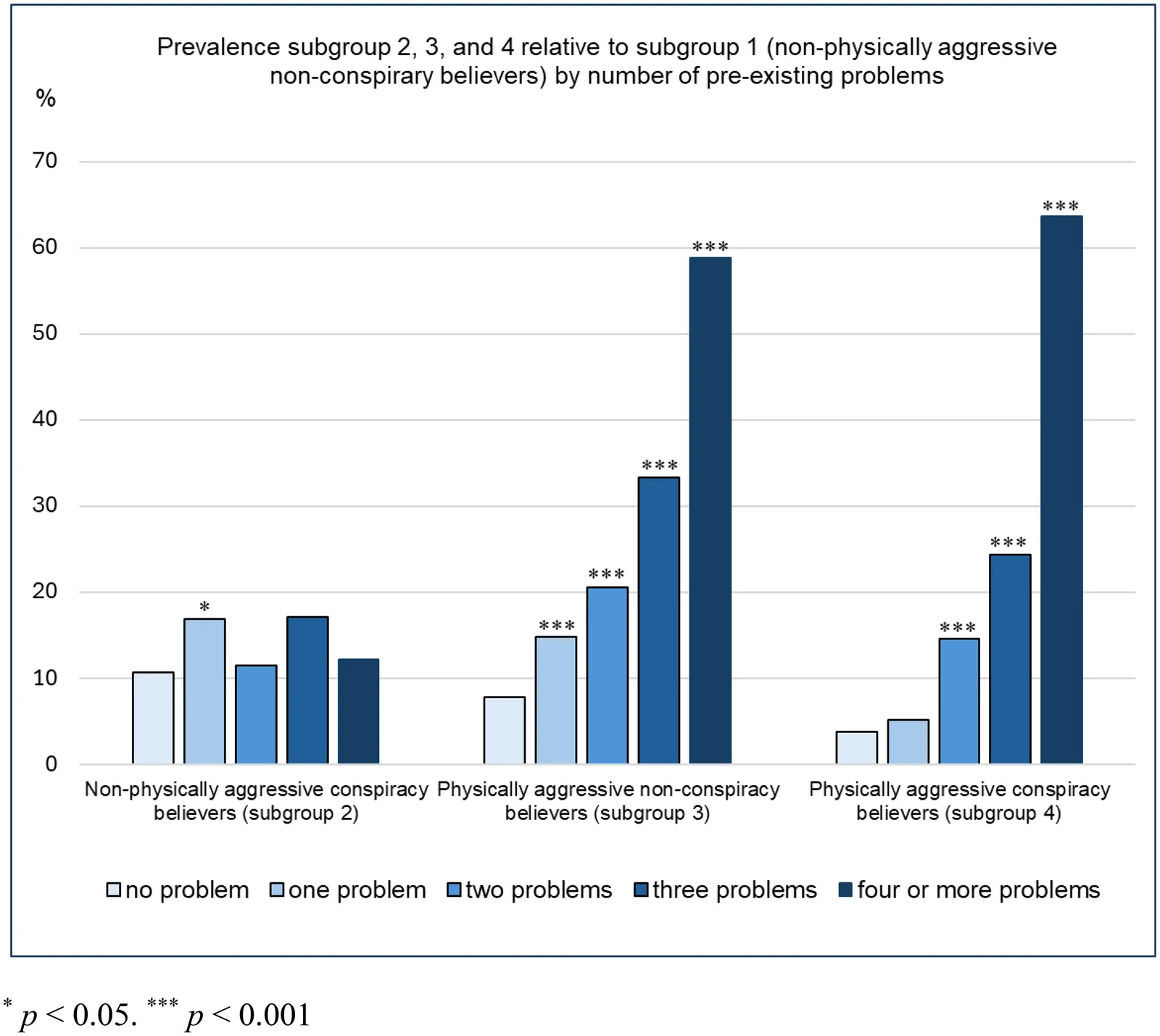
The number of pre-existing psychosocial problems and group membership.

**Fig 2:**
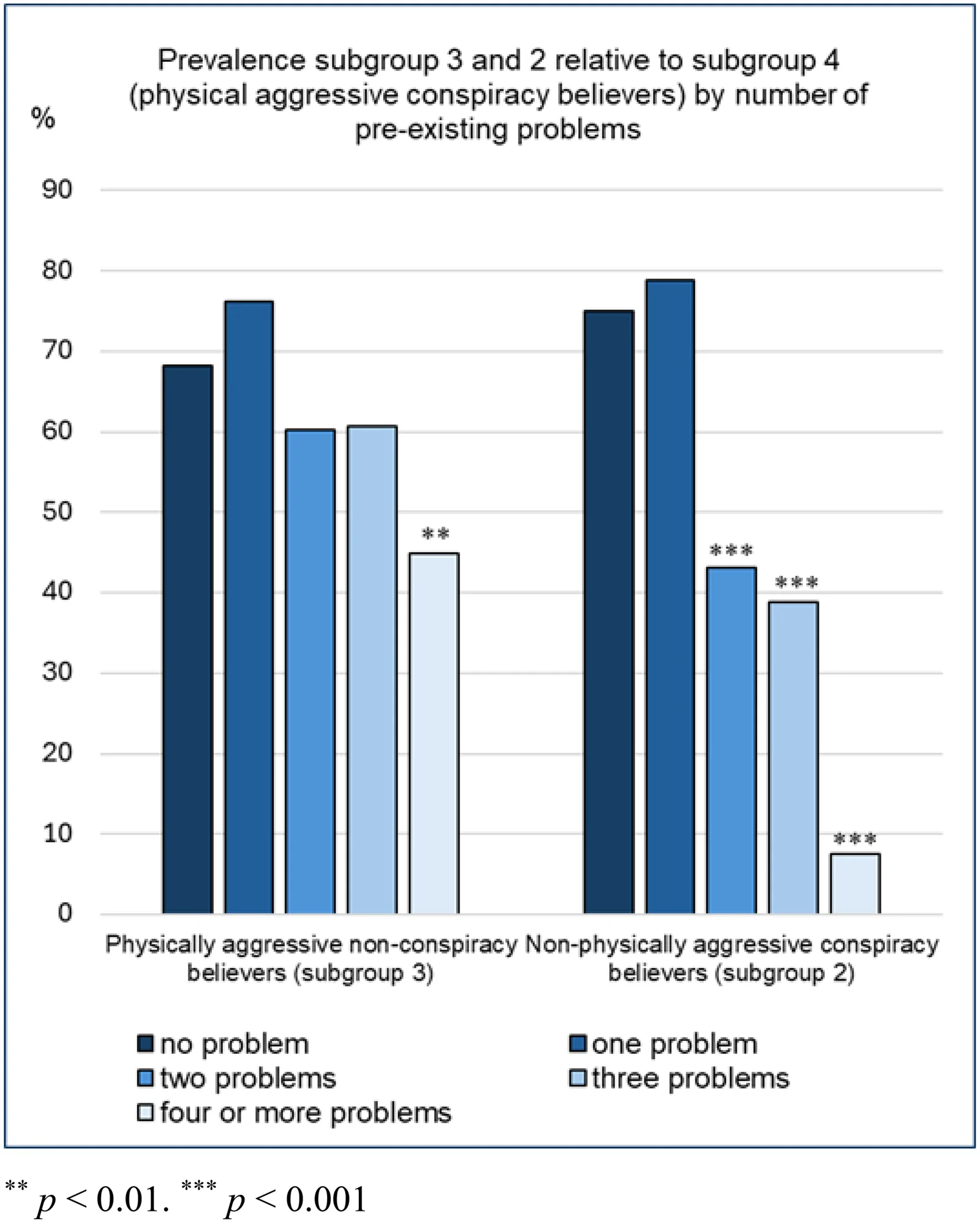
The number of pre-existing psychosocial problems and group membership.

In Figs 1 and 2 the results related to the *number* of pre-existing psychosocial problems are graphically illustrated. The asterisk in the figures refers to significant difference in prevalence of subgroups 2, 3 and 4 relative to subgroup 1 by the number of pre-existing psychosocial problems. Fig 1 for instance, shows that respondents with four or more pre-existing psychosocial problems significantly more often are physically aggressive conspiracy believers (63.9%) than those with no pre-existing psychosocial problems (3.8%) relative to non-physically aggressive non-conspiracy believers.

Control analyses showed no significant differences in total scores of conspiracy beliefs between non-physically aggressive believers (group 2) and physically aggressive conspiracy believers (group 4), and no significant differences in the total score on the aggression scale between physically aggressive non-conspiracy believers and physically aggressive conspiracy believers. Adding these two variables to the list of control variables did not affect the results. This indicates that the other observed differences between physically aggressive non-believers (group 3) and physically aggressive conspiracy believers (group 4) cannot be attributed to differences in physical aggression.

### Robustness of findings

The results with respect to cut-off scores of ≥ 5 (N^total^ = 1595) showed identical patterns across all 75 comparisons as the results based on the higher cut-off scores of ≥ 6 to identify groups, except two which were related to the non-physically aggressive groups 1 and 2 (see S3 and S4 Appendix). Respondents with one psychosocial problem were not significantly more often non-physically aggressive conspiracy believers than non-physically aggressive non-conspiracy believers (p^before correction^ = 0.047). In contrast, respondents with low life satisfaction were significantly more often non-physically aggressive conspiracy believers than non-physically aggressive non-conspiracy believers.

The results based on the analyses using the lower cut-off scores of ≥ 4 (N^total^ = 1903) showed almost identical patterns, also related to group 1 and 2 (see S5 and S6 Appendix). In contrast to the results using the cut-off scores ≥ 6, respondents with low life satisfaction were significantly more often non-physically aggressive conspiracy believers than non-physically aggressive non-conspiracy believers (aOR = 1.82, 95% CI = 1.18–2.81, p = 0.006). In addition, respondents with three psychosocial problems were significantly more often non-physically aggressive believers than non-physically aggressive non-believers (aOR = 1.80, 95% CI = 1.07–3.02, p = 0.026). Respondents with high ADS were not significantly more often physically aggressive believers than physically aggressive non-conspiracy believers. Respondents with very low life satisfaction were significantly less often physically aggressive non-believers than physically aggressive believers (aOR = 0.43, 95% CI = 0.27–0.68, p < 0.001).

Preliminary analyses showed that higher cut-off scores of ≥ 7 reflecting 6–7% highest scores, resulted in low counts in especially the subgroup of physically aggressive conspiracy believers (n = 79). We therefore did not repeat the MMLR analyses using higher cut-off scores.

## Discussion

In the present longitudinal population-based study we tested to following five hypotheses: respondents with pre-existing problems and a higher number of pre-existing problems are more often non-physically aggressive conspiracy believers (H1), more often physically aggressive non-conspiracy believers (H2), and more often physically aggressive conspiracy believers (H4) than non-physically aggressive non-conspiracy believers. In addition, we tested the hypotheses that respondents with pre-existing problems and a higher number of pre-existing problems are less often physically aggressive non-conspiracy believers (H4) and less often non-physically aggressive conspiracy believers (H5) than physically aggressive conspiracy believers. We focused on the following six pre-existing problems and the number of these problems: anxiety and depression symptoms, lack emotional support, exposure to traumatic and stressful life events, financial problems, and low life-satisfaction.

Based on the result the following main conclusions can be drawn. Among non-physically aggressive adults, those with pre-existing problems or a higher number of these problems were not more often conspiracy believers than those without pre-existing psycho-social problems. In other words, if individuals are not physically aggressive, pre-existing problems do not increase the likelihood that respondents are conspiracy believers rejecting H1. Adults with pre-existing psycho-social problems are much more often physically aggressive non-conspiracy believers and physically aggressive conspiracy believers than adults without these problems confirming H2 and H3. The pattern was observed for all assessed pre-existing problems and especially for the number of pre-existing problems: of those without any of the assessed problems 7.9% was an physically aggressive non-conspiracy believer and 3.8% a physically aggressive conspiracy believer while of those with four or more pre-existing problems 58.8% and 63.6% was a physically aggressive non-conspiracy believer and a physically aggressive conspiracy believer respectively, relative to non-physically aggressive non-conspiracy. The last findings, that 63.6% and 58.8% of those with four or more problems were physically aggressive respondents compared to non-physically aggressive non-conspiracy, suggest that adults with four or more pre-existing psycho-social problems are especially more likely to be physically aggressive, regardless of being a conspiracy believer or not. Importantly, findings clearly indicate that this is not the case: respondents with pre-existing problems were more often physically aggressive conspiracy believers than physically aggressive non-conspiracy believers, conforming H4. However, stressful life events and life-satisfaction did not discriminate between physically aggressive conspiracy believers and physically aggressive non-conspiracy believers. Nevertheless, in line with H5 those with (4 or more) pre-existing problems were more often physically aggressive conspiracy believers than non-physically aggressive conspiracy believers.

To test the robustness of our findings, we repeated the MMLR analyses using lower cut-off scores to identify physically aggressive respondents and conspiracy believers. Importantly, the outcomes showed identical results with minimal differences providing strong evidence for the observed patterns. Respondents who did not endorse any item of the physical aggression scale were safely considered not to be physically aggressive and who did not endorse any item of conspiracy scale were safely considered not to be a conspiracy believer.

To the best of our knowledge, there are no studies using a similar analytic strategy to compare our findings with. However, our results are in line with previous research [14–23]. The findings of our study are in line with theoretical models like the 3N model of Kruglanski that poses that aggression and conspiracy thinking have a motivational function in the way that they can restore feelings of self-worth of individuals who’s need for significance, which is the need to feel that one matters, merits respect and has social worth, is threatened [24]. However, our findings clearly showed that respondents with the assessed psychosocial problems or a higher number of these problems were more often physically aggressive than conspiracy believers. Importantly, among the non-physically aggressive subgroups those with these problems were *not* more often conspiracy believers. This may be why the well-known frustration-aggression hypothesis [19,40] offers a better explanation for physically aggressive behavior than the 3N model. Psychosocial problems thus seem to lead primarily to aggression due to frustration, and to a lesser extent to conspiracy thinking as a way of coping with this frustration.

The question is which need this belief addresses. It's possible that this belief aligns with less stress-related needs, such as excitement and entertainment [41].

Our findings clearly suggest that future studies on risk factors of conspiracy beliefs should distinguish believers who are and who are not physically aggressive.

Although multiple pre-existing psychosocial problems are strongly associated with being a physically aggressive conspiracy believer, a relatively small proportion (3.8%) of those without the assessed pre-existing problems were physically aggressive conspiracy believers. In other words, among those without any of the assessed pre-existing problems, physically aggressive conspiracy believers were not totally absent. This suggests that we should not automatically consider or treat physically aggressive conspiracy believers as one more or less homogenous group [41]. Although our research shows that psychosocial problems are associated with aggression and conspiracy thinking, there are also other factors that may play a role, such as personality factors [42,43]. These offer a possible explanation for the association between aggression and conspiracy thinking in individuals without psychosocial problems. The well-known frustration-aggression hypothesis and 3N model may therefore not apply to all physically aggressive conspiracy believers, but particularly to those with (multiple) pre-existing problems that fueled their frustrations. Similarly, beliefs in conspiracy theories may not without reservation be viewed as a way of providing a sense of meaning and purpose, or as a way to restore affected feelings of self-worth [9,44–47].

### Strengths and limitations

Major strengths of the present study are the use of a large representative sample and weighting of the data to optimize the representativeness, the use of validated measures, the longitudinal study design preventing the use of potentially biased retrospectively collected data on pre-existing psycho-social problems, controlling for relevant confounders, and applying the Benjamini-Hochberg correction of p-values [39] in the MMLR analyses using different cut-off scores.

However, the following limitations need to be clarified. In the present study we examined six different pre-existing problems but problems the participants may have faced are not limited to these six problems, such as discrimination and marginalization, and feelings of injustice and unresolved legal problems (apart from the consumption of false narratives in the (social) media such as about the “stolen elections”). These psychosocial problems can also trigger or evoke narratives other than conspiracy theories. Future studies are warranted to examine the extent to which other problems are (stronger) associated with being a (physically) aggressive) conspiracy believer. In addition, physical aggression and conspiracy beliefs were examined once and longitudinal data on physical aggression and conspiracy beliefs are needed to gain insight into the (causal) role of pre-existing problems in the course of physical aggression and conspiracy beliefs on the short and longer term (as well as bi-directional cross-lagged relationships). This could answer relevant questions such as to what extent adults who recovered from one or more pre-existing problems change in physical aggression and in conspiracy beliefs. To the best of our knowledge, to date longitudinal multi-wave studies on physical aggression and conspiracy beliefs among the general population are absent. Furthermore, although we examined the extent to which a higher number of pre-existing problems is associated with a higher likelihood of being a (aggressive) conspiracy believer, it was outside the aim of the present study to explore if and which specific combinations of pre-existing problems further increase the likelihood being a (aggressive) conspiracy believer. In addition, it was outside the aim of the present study to examine the extent to which other factors such as political ideology, partisanship, media exposure, and institutional trust play a role in the associations between existing problems and group membership.

The study by Enders and colleagues [48] showed that as the proportion of individuals believing a given conspiracy theory increases (from “holocaust deaths overrated” to “mail-in ballots increase fraud”), the weaker the connection between belief in that theory and support for (political) violence (p. 6). Our results are based on the 15-items Generic Conspiracist Beliefs Scale. Therefore, future studies are warranted to examine if studies using other questionnaires on conspiracy beliefs and/or on specific conspiracy theories such as the 12-item Conspiracy Mentality Scale (CMS) [49], the 1-Item Conspiracy Belief Scale (SCBS) [50], 8-item Beliefs in Conspiracy Theories (BCT) [51], and the 5-item Conspiracy Mentality Questionnaire (CMQ) [52] confirm our results (cf. [53]). In other words, conspiracy theory belief is not a single, uniform construct and specific beliefs may be related or shaped by different pre-existing psychosocial problems or factors [54].

To test the hypotheses, multivariate multinomial logistic regression (MMLR) analyses were conducted with group membership as dichotomous dependent variables (1–2, 1–3, 1–4, 4–3, 4–2). In the first series of MMLR analyses the number of respondents the aORs presented in Table 2 were derived from (besides control variables), ranged from 426 to 1091 (see S7 Appendix 7). In the second series of MMLR analyses the computed aORs presented in Table 3 were derived from smaller numbers of respondents, ranging from 64 to 326 with the dependent variable based on group 4 and group 3 membership, and 75–268 respondents with the dependent variable based on group 4 and group 2 membership. Especially the aORs related to life satisfaction and the number of psychosocial problems were derived from relatively small numbers (n ≤ 153) of respondents (see S7 Appendix 7) which may reduce the power of the MMLR analyses. This indicates that future studies with larger study samples are needed to confirm the findings related to these two variables. However, the additional analyses using lower cut-offs resulted in (much) higher numbers of respondents the aORs were derived from (See S7 Appendix 7) and showed similar patterns (the number of respondents from which the aORs were derived using the cut-off of ≥ 4 ranged from 173–589). We therefore believe that it is not very likely that larger studies will reveal different patterns. Given the critics on post-hoc power analysis [55] we chose not to conduct such analyses. In addition, the tables 2 and 3, and appendix 1–6 show that the event-per-variable ratios are all above 10 [56].

This study was conducted in a Western country (the Netherlands). We may expect that part of the adult residents of other countries faced similar psychosocial problems, although the prevalence may differ. Future studies in other Western and non-Western countries using a similar study design are warranted because they provide insight into the extent to which societal characteristics (such as economic development and employment rates; health, legal and social systems) weaken or strengthen the associations between psychosocial problems on the one hand and conspiracy beliefs and physically aggressive behavior on the other.

Finally, it was outside the aim of the present study to examine the effects of being a (physically aggressive) conspiracy believer or group membership on for instance (future) political engagement and political participation [57,58].

### Practical relevance

An important novel finding of the present study is that respondents with assessed pre-existing psychosocial problems and a higher number of these problems, indicative of very unfavorable life conditions, considerably more often are physically aggressive conspiracy believers than non-physically aggressive non-conspiracy believers. In contrast, respondents with assessed pre-existing psychosocial problems and a higher number of these problems hardly differentiate between non-physically aggressive non-conspiracy believers and non-physically aggressive conspiracy believers: those with problems are not systematically more often non-physically aggressive conspiracy believers than non-physically aggressive non-conspiracy believers.

Results suggest that policies and interventions to reduce the adoption and dispersion of conspiracy beliefs combined with physical aggression should in any case, besides other potential measures [59], be aimed at preventing and reducing these pre-existing problems [9,44]. Given the strong relationship between the number of different pre-existing problems and being a physically aggressive conspiracy believer, this may require a multidisciplinary approach. This could involve addressing mental health problems (for example by securing mental health care access), ánd financial problems (for example by organizing governmental support) ánd prevention of violence (for example by investments in public security).

## Supporting information

S1 Appendix 1. Results multivariate multinomial logistic regression analysis Part 1 with cut-offs ≥6. (DOCX)

S2 Appendix 2. Results multivariate multinomial logistic regression analysis Part 2 with cut-offs ≥6. (DOCX)

S3 Appendix 3. Results multivariate multinomial logistic regression analysis Part 1 with cut-offs ≥5. (DOCX)

S4 Appendix 4. Results multivariate multinomial logistic regression analysis Part 2 with cut-offs ≥5. (DOCX)

S5 Appendix 5. Results multivariate multinomial logistic regression analysis Part 1 with cut-offs ≥4. (DOCX)

S6 Appendix 6. Results multivariate multinomial logistic regression analysis Part 2 with cut-offs ≥4. (DOCX)

S7 Appendix 7. Number respondents AOR were derived from. (DOCX)
