# Supplementary material for: Physically aggressive conspiracy believers often faced various psycho-social problems: Findings from a longitudinal population-based study
Source: PLoS One. 2026 Sep 9;21(9):e0353935. doi: 10.1371/journal.pone.0353935 (PMC13557364; doi:10.1371/journal.pone.0353935)
Supplement: Supplementary file 1 [file pone.0353935.s001.docx]

**S1 Appendix 1.** Results multivariate multinomial logistic regression analysis Part 1 with cut-offs ≥ 6

|  | Adjusted OR (95% CI) relative to the *Non-physical aggressive non-conspiracy believers* group (Group 1) | | | | | |  |
| --- | --- | --- | --- | --- | --- | --- | --- |
|  | Group 2: *Non-physical aggressive conspiracy believers* |  | Group 3: *Physical aggressive non-conspiracy believers* |  | Group 4: *Physical aggressive conspiracy believers* |  |  |
|  | AOR (95% CI) | p | AOR (95% CI) | p | AOR (95% CI) | p |  |
| Anxiety and depression symptoms levels (T1a) | | | | | |  |  |
| - no (ref.) | 1 |  | 1 |  | 1 |  |  |
| - moderate | 0.64 (0.35-1.16) | 0.139 | 3.65 (2.49-5.36) | 0.000 | 3.66 (2.28-5.89) | 0.000 |  |
| - high | 0.49 (0.14-1.75) | 0.271 | 9.40 (5.29-16.70) | 0.000 | 19.81 (10.88-36.08) | 0.000 |  |
| Lack of emotional support (T1a) | | | | | |  |  |
| - no (ref.) | 1 |  | 1 |  | 1 |  |  |
| - medium | 1.12 (0.69-1.79) | 0.653 | 2.03 (1.36-3.03) | 0.000 | 2.92 (1.77-4.82) | 0.000 |  |
| - high | 1.11 (0.62-1.98) | 0.727 | 3.66 (2.39-5.61) | 0.000 | 8.95 (5.59-14.32) | 0.000 |  |
| Potential traumatic events (in 12 months before T1a) | | | | | |  |  |
| - no (ref.) | 1 |  | 1 |  | 1 |  |  |
| - yes | 0.90 (0.36-2.27) | 0.825 | 3.55 (2.09-6.03) | 0.000 | 9.25 (5.55-15.43) | 0.000 |  |
| Stressful life-events (in 12 months before T1a) | | | | | |  |  |
| - no (ref.) | 1 |  | 1 |  | 1 |  |  |
| - yes | 1.01 (0.66-1.54) | 0.971 | 2.37 (1.64-3.41) | 0.000 | 3.10 (2.05-4.69) | 0.000 |  |
| Financial problems (T1a) | | | | | |  |  |
| - no (ref.) | 1 |  | 1 |  | 1 |  |  |
| - yes | 1.16 (0.41-3.29) | 0.774 | 5.21 (2.67-10.15) | 0.000 | 16.13 (8.50-30.63) | 0.000 |  |
| Life satisfaction (T1b) | | | | | | | |
| - very high (ref.) | 1 |  | 1 |  | 1 |  |  |
| - high | 0.91 (0.50-1.67) | 0.765 | 1.16 (0.61-2.21) | 0.656 | 0.98 (0.39-2.48) | 0.966 |  |
| - medium | 1.10 (0.64-1.88) | 0.730 | 2.79 (1.64-4.74) | 0.000 | 3.00 (1.48-6.10) | 0.002 |  |
| - low | 1.74 (1.01-3.00) | 0.046^1^ | 4.01 (2.35-6.86) | 0.000 | 4.45 (2.20-9.00) | 0.000 |  |
| - very low | 1.54 (0.89-2.66) | 0.121 | 4.74 (2.78-8.07) | 0.000 | 10.45 (5.42-20.13) | 0.000 |  |
| Number of assessed problems | | | | | | | |
| - zero (ref.) | 1 |  | 1 |  | 1 |  |  |
| - one | 1.62 (1.05-2.50) | 0.030 | 2.39 (1.45-3.93) | 0.001 | 1.64 (0.77-3.50) | 0.198 |  |
| - two | 1.03 (0.59-1.79) | 0.927 | 3.47 (2.07-5.82) | 0.000 | 4.78 (2.43-9.41) | 0.000 |  |
| - three | 1.61 (0.83-3.12) | 0.158 | 7.35 (4.11-13.16) | 0.000 | 10.13 (4.82-21.26) | 0.000 |  |
| - four or more | 1.20 (0.41-3.46) | 0.740 | 20.28 (10.52-39.11) | 0.000 | 58.10 (27.25-123.86) | 0.000 |  |

^1^ Not significant after the Benjamini-Hochberg correction of p-values.
