# Supplementary material for: Physically aggressive conspiracy believers often faced various psycho-social problems: Findings from a longitudinal population-based study
Source: PLoS One. 2026 Sep 9;21(9):e0353935. doi: 10.1371/journal.pone.0353935 (PMC13557364; doi:10.1371/journal.pone.0353935)
Supplement: Supplementary file 2 [file pone.0353935.s002.docx]

**S2 Appendix 2.** Results multivariate multinomial logistic regression analysis Part 2 with cut-offs ≥ 6

|  | Adjusted OR (95% CI) relative to P*hysical aggressive conspiracy believers* group (Group 4) | | | |
| --- | --- | --- | --- | --- |
|  | Group 3: | | Group 2: | |
|  | *Physical aggressive non-conspiracy believers* | | *Non-physical aggressive conspiracy believers* | |
|  | AOR (95% CI) | p | AOR (95% CI) | p |
|  |  |  |  |  |
| Anxiety and depression symptoms levels (T1a) | | | |  |
| - no (ref.) | 1 |  | 1 |  |
| - moderate | 1.00 (0.59-1.70) | 0.994 | 0.17 (0.09-0.36) | 0.000 |
| - high | 0.47 (0.26-0.86) | 0.014 | 0.02 (0.01-0.09) | 0.000 |
| Lack of emotional support (T1a) | |  |  |  |
| - no (ref.) | 1 |  | 1 |  |
| - medium | 0.70 (0.39-1.24) | 0.217 | 0.38 (0.20-0.73) | 0.003 |
| - high | 0.41 (0.24-0.69) | 0.001 | 0.12 (0.06-0.25) | 0.000 |
| Potential traumatic events (in 12 months before T1a) | | | |  |
| - no (ref.) | 1 |  | 1 |  |
| - yes | 0.38 (0.22-0.67) | 0.001 | 0.10 (0.04-0.25) | 0.000 |
| Stressful life-events (in 12 months before T1a) | | | |  |
| - no (ref.) | 1 |  | 1 |  |
| - yes | 0.76 (0.47-1.23) | 0.271 | 0.33 (0.19-0.57) | 0.000 |
| Financial problems (T1a) | |  |  |  |
| - no (ref.) | 1 |  | 1 |  |
| - yes | 0.32 (0.17-0.61) | 0.000 | 0.07 (0.02-0.21) | 0.000 |
| Life satisfaction (T1b) | |  |  |  |
| - very high (ref.) | 1 |  | 1 |  |
| - high | 1.18 (0.40-3.49) | 0.763 | 0.93 (0.32-2.73) | 0.896 |
| - medium | 0.93 (0.41-2.13) | 0.862 | 0.37 (0.16-0.86) | 0.021 |
| - low | 0.90 (0.40-2.05) | 0.806 | 0.39 (0.17-0.91) | 0.029 |
| - very low | 0.45 (0.21-0.98) | 0.045^1^ | 0.15 (0.07-0.33) | 0.000 |
| Number of assessed problems | |  |  |  |
| - zero (ref.) | 1 |  | 1 |  |
| - one | 1.45 (0.61-3.45) | 0.397 | 0.98 (0.42-2.29) | 0.971 |
| - two | 0.73 (0.33-1.61) | 0.433 | 0.21 (0.09-0.50) | 0.000 |
| - three | 0.73 (0.31-1.68) | 0.451 | 0.16 (0.06-0.40) | 0.000 |
| - four or more | 0.35 (0.16-0.77) | 0.009 | 0.02 (0.01-0.07) | 0.000 |

^1^ Not significant after the Benjamini-Hochberg correction of p-values.
