# Supplementary material for: Physically aggressive conspiracy believers often faced various psycho-social problems: Findings from a longitudinal population-based study
Source: PLoS One. 2026 Sep 9;21(9):e0353935. doi: 10.1371/journal.pone.0353935 (PMC13557364; doi:10.1371/journal.pone.0353935)
Supplement: Supplementary file 3 [file pone.0353935.s003.docx]

**S3 Appendix 3.** Results multivariate multinomial logistic regression analysis Part 1 with cut-offs ≥ 5

|  |  |  | Adjusted OR (95% CI) relative to the *Not physical aggressive, has No conspiracy beliefs* group (Group 1) | | | | | |  |  |  |  |  |
| --- | --- | --- | --- | --- | --- | --- | --- | --- | --- | --- | --- | --- | --- |
|  |  | Group 1:  *Non-physical aggressive non-conspiracy believers* (n=894) | Group 2: *Non-physical aggressive conspiracy believers* (n=184) | | Group 3: *Physical aggressive non-conspiracy believers* (n=292) | | Group 4: *Physical aggressive conspiracy believers* (n=224) | |  |  |  |  |  |
|  | N^total^ |  | n (%) | AOR (95% CI) | n (%) | AOR (95% CI) | n (%) | AOR (95% CI) |  |  |  |  |  |
| Anxiety and depression symptoms levels (T1a) | | | | | | | |  |  |  |  |  |  |
| - no (ref.) | 1175 | 741 (82.2 / 82.3 / 86.7) | 161 (17.8) | 1 | 159 (17.7) | 1 | 114 (13.3) | 1 |  |  |  |  |  |
| - moderate | 290 | 125 (88.0 / 58.7 / 67.6) | 17 (12.0) | 0.60 (0.34-1.03) | 88 (41.3) | 2.97 (2.12-4.16)^***^ | 60 (32.4) | 2.89 (1.98-4.21)^***^ |  |  |  |  |  |
| - high | 131 | 28 (80.0 / 37.8 / 35.9) | 7 (20.0) | 1.00 (0.42-2.36) | 46 (62.2) | 7.37 (4.37-12.43)^***^ | 50 (64.1) | 11.42 (6.74-19.35)^***^ |  |  |  |  |  |
| Lack of emotional support (T1a) | | | | | | | |  |  |  |  |  |  |
| - no (ref.) | 1009 | 649 (83.9 / 81.4 / 88.2) | 125 (16.1) | 1 | 148 (18.6) | 1 | 87 (11.8) | 1 |  |  |  |  |  |
| - medium | 317 | 154 (81.9 / 67.8 / 73.3) | 34 (18.1) | 1.19 (0.78-1.82) | 73 (32.2) | 1.95 (1.38-2.74)^***^ | 56 (26.7) | 2.55 (1.73-3.76)^***^ |  |  |  |  |  |
| - high | 267 | 90 (78.3 / 55.9 / 52.6) | 25 (21.7) | 1.41 (0.87-2.30) | 71 (44.1) | 3.43 (2.37-4.97)^***^ | 81 (47.4) | 6.64 (4.51-9.76)^***^ |  |  |  |  |  |
| Potential traumatic events (in 12 months before T1a) | | | | | | | |  |  |  |  |  |  |
| - no (ref.) | 1453 | 855 (82.8 / 77.2 / 83.7) | 178 (17.2) | 1 | 253 (22.8) | 1 | 167 (16.3) | 1 |  |  |  |  |  |
| - yes | 141 | 39 (86.7 / 50.0 / 40.6) | 6 (13.3) | 0.76 (0.31-1.81) | 39 (50.0) | 3.23 (1.99-5.22)^***^ | 57 (59.4) | 7.18 (4.55-11.34)^***^ |  |  |  |  |  |
| Stressful life-events (in 12 months before T1a) | | | | | | | |  |  |  |  |  |  |
| - no (ref.) | 1138 | 669 (83.1 / 77.2 / 83.2) | 136 (16.9) | 1 | 198 (22.8) | 1 | 135 (16.8) | 1 |  |  |  |  |  |
| - yes | 455 | 224 (82.4 / 70.4 / 71.6) | 48 (17.6) | 1.07 (0.73-1.55) | 94 (29.6) | 2.09 (1.53-2.86)^***^ | 89 (28.4) | 2.84 (2.04-3.97)^***^ |  |  |  |  |  |
| Financial problems (T1a) | | | | | | | |  |  |  |  |  |  |
| - no (ref.) | 1494 | 873 (83.3 / 76.7 / 82.8) | 175 (16.7) | 1 | 265 (23.3) | 1 | 181 (17.2) | 1 |  |  |  |  |  |
| - yes | 100 | 21 (70.0 / 43.8 / 32.8) | 9 (30.0) | 1.93 (0.87-4.29) | 27 (56.3) | 4.39 (2.38-8.09)^***^ | 43 (67.2) | 10.85 (6.09-19.31)^***^ |  |  |  |  |  |
| Life satisfaction (T1b) | |  |  |  |  |  |  |  |  |  |  |  |  |
| - very high (ref.) | 419 | 298 (85.9 / 86.4 / 92.3) | 49 (14.1) | 1 | 47 (13.6) | 1 | 25 (7.7) | 1 |  |  |  |  |  |
| - high | 231 | 159 (86.4 / 83.7 / 90.9) | 25 (13.6) | 0.99 (0.58-1.67) | 31 (16.3) | 1.18 (0.71-1.95) | 16 (9.1) | 1.15 (0.60-2.23) |  |  |  |  |  |
| - medium | 340 | 179 (82.9 / 70.8 / 78.2) | 37 (17.1) | 1.18 (0.73-1.90) | 74 (29.2) | 2.44 (1.59-3.74)^***^ | 50 (21.8) | 3.10 (1.83-5.23)^***^ |  |  |  |  |  |
| - low | 268 | 125 (77.6 / 66.8 / 73.5) | 36 (22.4) | 1.79 (1.10-2.92)^*^ | 62 (33.2) | 3.00 (1.92-4.69)^***^ | 45 (26.5) | 4.08 (2.38-7.01)^***^ |  |  |  |  |  |
| - very low | 337 | 133 (77.8 / 62.7 / 60.5) | 38 (22.2) | 1.59 (0.98-2.59) | 79 (37.3) | 3.96 (2.56-6.15)^***^ | 87 (39.5) | 8.25 (4.96-13.73)^***^ |  |  |  |  |  |
| Number of assessed problems | | | | | | | | |  |  |  |  |  |
| - zero (ref.) | 487 | 352 (86.1 / 88.0 / 92.1) | 57 (13.9) | 1 | 48 (12.0) | 1 | 30 (7.9) | 1 |  |  |  |  |  |
| - one | 452 | 276 (80.2 / 78.6 / 89.3) | 68 (19.8) | 1.49 (1.01-2.20)^*1^ | 75 (21.4) | 2.27 (1.51-3.42)^***^ | 33 (10.7) | 1.59 (0.94-2.71) |  |  |  |  |  |
| - two | 328 | 170 (83.3 / 71.1 / 75.6) | 34 (16.7) | 1.22 (0.77-1.95) | 69 (28.9) | 3.18 (2.08-4.86)^***^ | 55 (24.4) | 4.03 (2.46-6.60)^***^ |  |  |  |  |  |
| - three | 187 | 68 (79.1 / 54.4 / 60.7) | 18 (20.9) | 1.56 (0.86-2.83) | 57 (45.6) | 7.17 (4.41-11.65)^***^ | 44 (39.3) | 9.03 (5.20-15.67)^***^ |  |  |  |  |  |
| - four or more | 143 | 28 (77.8 / 38.9 / 30.8) | 8 (22.2) | 1.65 (0.70-3.89) | 44 (61.1) | 13.73 (7.58-24.85)^***^ | 63 (69.2) | 32.77 (17.69-60.68)^***^ |  |  |  |  |  |

^1^ Not significant after the Benjamini-Hochberg correction of p-values. ^*^ *p* < 0.05, ^**^ *p* < 0.01, ^***^ *p* < 0.001
