# Supplementary material for: Physically aggressive conspiracy believers often faced various psycho-social problems: Findings from a longitudinal population-based study
Source: PLoS One. 2026 Sep 9;21(9):e0353935. doi: 10.1371/journal.pone.0353935 (PMC13557364; doi:10.1371/journal.pone.0353935)
Supplement: Supplementary file 4 [file pone.0353935.s004.docx]

**S4 Appendix 4.** Results multivariate multinomial logistic regression analysis Part 2 with cut-offs ≥ 5

|  |  |  | Adjusted OR (95% CI) relative to P*hysical aggressive. has conspiracy beliefs* group (Group 4) | | | |  |  |  |  |  |  |  |
| --- | --- | --- | --- | --- | --- | --- | --- | --- | --- | --- | --- | --- | --- |
|  |  | Group 4: *Physical aggressive conspiracy believers* (n=224) | Group 3: *Physical aggressive non-conspiracy believers* (n=292) | | Group 2: *Non-physical aggressive conspiracy believers* (n=184) | |  |  |  |  |  |  |  |
|  | N^total^ |  | n (%) | AOR (95% CI) | n (%) | AOR (95% CI) |  |  |  |  |  |  |  |
| Anxiety and depression symptoms levels (T1a) | | | | | | | | | |  |  |  |  |
| - no (ref.) | 1175 | 114 (41.8 / 41.5) | 159 (58.2) | 1 | 161 (58.5) | 1 |  |  |  |  |  |  |  |
| - moderate | 290 | 60 (40.5 / 77.9) | 88 (59.5) | 1.03 (0.68-1.56) | 17 (22.1) | 0.21 (0.11-0.38)^***^ |  |  |  |  |  |  |  |
| - high | 131 | 50 (52.1 / 87.7) | 46 (47.9) | 0.65 (0.40-1.05) | 7 (12.3) | 0.09 (0.04-0.21)^***^ |  |  |  |  |  |  |  |
| Lack of emotional support (T1a) | | | | | | | | | |  |  |  |  |
| - no (ref.) | 1009 | 87 (37.0 / 41.0) | 148 (63.0) | 1 | 125 (59.0) | 1 |  |  |  |  |  |  |  |
| - medium | 317 | 56 (43.4 / 62.2) | 73 (56.6) | 0.76 (0.49-1.19) | 34 (37.8) | 0.47 (0.28-0.79)^**^ |  |  |  |  |  |  |  |
| - high | 267 | 81 (53.3 / 76.4) | 71 (46.7) | 0.52 (0.34-0.78)^**^ | 25 (23.6) | 0.21 (0.12-0.36)^***^ |  |  |  |  |  |  |  |
| Potential traumatic events (in 12 months before T1a) | | | | | | | | | |  |  |  |  |
| - no (ref.) | 1245 | 167 (39.8 / 48.4) | 253 (60.2) | 1 | 178 (51.6) | 1 |  |  |  |  |  |  |  |
| - yes | 112 | 57 (59.4 / 90.5) | 39 (40.6) | 0.45 (0.28-0.71)^***^ | 6 (9.5) | 0.11 (0.04-0.25)^***^ |  |  |  |  |  |  |  |
| Stressful life events (in 12 months before T1a) | | | | | | | | | |  |  |  |  |
| - no (ref.) | 1138 | 135 (40.5 / 49.8) | 198 (59.5) | 1 | 136 (50.2) | 1 |  |  |  |  |  |  |  |
| - yes | 455 | 89 (48.6 / 65.0) | 94 (51.4) | 0.74 (0.50-1.07) | 48 (35.0) | 0.37 (0.24-0.59)^***^ |  |  |  |  |  |  |  |
| Financial problems (T1a) | |  |  |  |  |  |  |  |  |  |  |  |  |
| - no (ref.) | 1494 | 181 (40.6 / 50.8) | 265 (59.4) | 1 | 175 (49.2) | 1 |  |  |  |  |  |  |  |
| - yes | 100 | 43 (61.4 / 82.7) | 27 (38.6) | 0.40 (0.24-0.69)^***^ | 9 (17.3) | 0.18 (0.08-0.39)^***^ |  |  |  |  |  |  |  |
| Life satisfaction (T1b) |  |  |  |  |  |  |  |  |  |  |  |  |  |
| - very high (ref.) | 419 | 25 (34.7 / 33.8) | 47 (65.3) | 1 | 49 (66.2) | 1 |  |  |  |  |  |  |  |
| - high | 231 | 16 (34.0 / 39.0) | 31 (66.0) | 1.02 (0.47-2.20) | 25 (61.0) | 0.85 (0.38-1.90) |  |  |  |  |  |  |  |
| - medium | 340 | 50 (40.3 / 57.5) | 74 (59.7) | 0.79 (0.43-1.45) | 37 (42.5) | 0.38 (0.20-0.74)^**^ |  |  |  |  |  |  |  |
| - low | 268 | 45 (42.1 / 55.6) | 62 (57.9) | 0.73 (0.39-1.37) | 36 (44.4) | 0.44 (0.23-0.86)^*^ |  |  |  |  |  |  |  |
| - very low | 337 | 87 (52.4 / 69.6) | 79 (47.6) | 0.48 (0.27-0.86)^*^ | 38 (30.4) | 0.19 (0.10-0.37)^***^ |  |  |  |  |  |  |  |
| Number of assessed problems | |  |  |  |  |  |  |  |  |  |  |  |  |
| - zero (ref.) | 487 | 30 (38.5 / 34.5) | 48 (61.5) | 1 | 57 (65.5) | 1 |  |  |  |  |  |  |  |
| - one | 452 | 33 (30.6 / 32.7) | 75 (69.4) | 1.43 (0.77-2.64) | 68 (67.3) | 0.93 (0.50-1.74) |  |  |  |  |  |  |  |
| - two | 328 | 55 (44.4 / 61.8) | 69 (55.6) | 0.79 (0.44-1.41) | 34 (38.2) | 0.30 (0.16-0.57)^***^ |  |  |  |  |  |  |  |
| - three | 187 | 44 (43.6 / 71.0) | 57 (56.4) | 0.79 (0.43-1.46) | 18 (29.0) | 0.17 (0.08-0.36)^***^ |  |  |  |  |  |  |  |
| - four or more | 143 | 63 (58.9 / 88.7) | 44 (41.1) | 0.42 (0.23-0.77)^**^ | 8 (11.3) | 0.05 (0.02-0.12)^***^ |  |  |  |  |  |  |  |

^1^ Not significant after the Benjamini-Hochberg correction of p-values. ^*^ *p* < 0.05. ^**^ *p* < 0.01. ^***^ *p* < 0.001
