# Supplementary material for: Physically aggressive conspiracy believers often faced various psycho-social problems: Findings from a longitudinal population-based study
Source: PLoS One. 2026 Sep 9;21(9):e0353935. doi: 10.1371/journal.pone.0353935 (PMC13557364; doi:10.1371/journal.pone.0353935)
Supplement: Supplementary file 5 [file pone.0353935.s005.docx]

**S4 Appendix 5.** Results multivariate multinomial logistic regression analysis Part 1 with cut-offs ≥ 4

|  |  |  | Adjusted OR (95% CI) relative to the *Not physical aggressive, has No conspiracy beliefs* group (Group 1) | | | | | |  |  |  |  |  |
| --- | --- | --- | --- | --- | --- | --- | --- | --- | --- | --- | --- | --- | --- |
|  |  | Group 1:  *Non-physical aggressive non-conspiracy believers* (n=894) | Group 2: Non-physical aggressive conspiracy believers (n=243) | | Group 3: *Physical aggressive non-conspiracy believers* (n=420) | | Group 4: *Physical aggressive conspiracy believers* (n=346) | |  |  |  |  |  |
|  | N^total^ |  | n (%) | AOR (95% CI) | n (%) | AOR (95% CI) | n (%) | AOR (95% CI) |  |  |  |  |  |
| Anxiety and depression symptoms levels (T1a) | | | | | | | |  |  |  |  |  |  |
| - no (ref.) | 1392 | 741 (77.9 / 74.8 / 79.5) | 210 (22.1) | 1 | 250 (25.2) | 1 | 191 (20.5) | 1 |  |  |  |  |  |
| - moderate | 355 | 125 (85.6 / 52.5 / 56.6) | 21 (14.4) | 0.60 (0.37-0.99)^*1^ | 113 (47.5) | 2.56 (1.89-3.47)^***^ | 96 (43.4) | 2.73 (1.98-3.77)^***^ |  |  |  |  |  |
| - high | 156 | 28 (70.0 / 32.9 / 32.2) | 12 (30.0) | 1.39 (0.68-2.84) | 57 (67.1) | 5.90 (3.62-9.61)^***^ | 59 (67.8) | 7.56 (4.63-12.36)^***^ |  |  |  |  |  |
| Lack of emotional support (T1a) | | | | | | | |  |  |  |  |  |  |
| - no (ref.) | 1173 | 649 (79.8 / 74.6 / 82.4) | 164 (20.2) | 1 | 221 (25.4) | 1 | 139 (17.6) | 1 |  |  |  |  |  |
| - medium | 404 | 154 (76.6 / 59.2 / 61.4) | 47 (23.4) | 1.26 (0.87-1.83) | 106 (40.8) | 1.92 (1.43-2.59)^***^ | 97 (38.6) | 2.80 (2.03-3.86)^***^ |  |  |  |  |  |
| - high | 325 | 90 (73.8 / 49.2 / 45.0) | 32 (26.2) | 1.38 (0.89-2.15) | 93 (50.8) | 2.99 (2.14-4.18)^***^ | 110 (55.0) | 5.56 (3.95-7.83)^***^ |  |  |  |  |  |
| Potential traumatic events (in 12 months before T1a) | | | | | | | |  |  |  |  |  |  |
| - no (ref.) | 1723 | 855 (78.8 / 69.8 / 76.1) | 230 (21.2) | 1 | 370 (30.2) | 1 | 268 (23.9) | 1 |  |  |  |  |  |
| - yes | 180 | 39 (75.0 / 43.8 / 33.3) | 13 (25.0) | 1.33 (0.70-2.54) | 50 (56.2) | 2.66 (1.70-4.15)^***^ | 78 (66.7) | 5.89 (3.88-8.94)^***^ |  |  |  |  |  |
| Stressful life events (in 12 months before T1a) | | | | | | | |  |  |  |  |  |  |
| - no (ref.) | 1335 | 669 (79.1 / 70.5 / 76.2) | 177 (20.9) | 1 | 280 (29.5) | 1 | 209 (23.8) | 1 |  |  |  |  |  |
| - yes | 567 | 224 (77.2 / 61.5 / 62.0) | 66 (22.8) | 1.14 (0.82-1.58) | 140 (38.5) | 1.97 (1.51-2.58)^***^ | 137 (38.0) | 2.65 (2.00-3.52)^***^ |  |  |  |  |  |
| Financial problems (T1a) | |  |  |  |  |  |  |  |  |  |  |  |  |
| - no (ref.) | 1783 | 873 (79.1 / 69.3 / 74.9) | 231 (20.9) | 1 | 387 (30.7) | 1 | 292 (25.1) | 1 |  |  |  |  |  |
| - yes | 120 | 21 (63.6 / 38.9 / 28.0) | 12 (36.4) | 1.93 (0.93-4.01) | 33 (61.1) | 3.59 (2.02-6.37)^***^ | 54 (72.0) | 7.41 (4.32-12.70)^***^ |  |  |  |  |  |
| Life satisfaction (T1b) | |  |  |  |  |  |  |  |  |  |  |  |  |
| - very high (ref.) | 487 | 298 (81.9 / 78.6 / 87.6) | 66 (18.1) | 1 | 81 (21.4) | 1 | 42 (12.4) | 1 |  |  |  |  |  |
| - high | 271 | 159 (82.4 / 77.2 / 83.7) | 34 (17.6) | 0.99 (0.62-1.58) | 47 (22.8) | 1.04 (0.69-1.58) | 31 (16.3) | 1.33 (0.80-2.22) |  |  |  |  |  |
| - medium | 405 | 179 (79.2 / 62.6 / 71.3) | 47 (20.8) | 1.12 (0.73-1.71) | 107 (37.4) | 2.18 (1.53-3.11)^***^ | 72 (28.7) | 2.76 (1.79-4.25)^***^ |  |  |  |  |  |
| - low | 332 | 125 (71.8 / 59.5 / 63.1) | 49 (28.2) | 1.82 (1.18-2.81)^**^ | 85 (40.5) | 2.52 (1.72-3.68)^***^ | 73 (36.9) | 4.01 (2.58-6.25)^***^ |  |  |  |  |  |
| - very low | 408 | 133 (73.9 / 57.1 / 51.0) | 47 (26.1) | 1.49 (0.96-2.32) | 100 (42.9) | 3.01 (2.06-4.38)^***^ | 128 (49.0) | 7.05 (4.62-10.75)^***^ |  |  |  |  |  |
| Number of assessed problems | | | | | | | | |  |  |  |  |  |
| - zero (ref.) | 550 | 352 (82.2 / 81.7 / 89.1) | 76 (17.8) | 1 | 79 (18.3) | 1 | 43 (10.9) | 1 |  |  |  |  |  |
| - one | 530 | 276 (76.7 / 71.7 / 81.9) | 84 (23.3) | 1.41 (0.99-2.01) | 109 (28.3) | 2.01 (1.43-2.81)^***^ | 61 (18.1) | 2.04 (1.33-3.14)^**^ |  |  |  |  |  |
| - two | 407 | 170 (78.7 / 62.7 / 65.4) | 46 (21.3) | 1.27 (0.84-1.92) | 101 (37.3) | 2.91 (2.04-4.16)^***^ | 90 (34.6) | 4.67 (3.08-7.08)^***^ |  |  |  |  |  |
| - three | 247 | 68 (71.6 / 45.9 / 48.6) | 27 (28.4) | 1.80 (1.07-3.02)^*^ | 80 (54.1) | 5.91 (3.88-9.00)^***^ | 72 (51.4) | 9.60 (5.99-15.41)^***^ |  |  |  |  |  |
| - four or more | 171 | 28 (71.8 / 35.0 / 25.9) | 11 (28.2) | 1.82 (0.85-3.88) | 52 (65.0) | 9.39 (5.46-16.13)^***^ | 80 (74.1) | 26.48 (15.16-46.24)^***^ |  |  |  |  |  |

^1^ Not significant after the Benjamini-Hochberg correction of p-values. ^*^ *p* < 0.05, ^**^ *p* < 0.01, ^***^ *p* < 0.001
