# Supplementary material for: Physically aggressive conspiracy believers often faced various psycho-social problems: Findings from a longitudinal population-based study
Source: PLoS One. 2026 Sep 9;21(9):e0353935. doi: 10.1371/journal.pone.0353935 (PMC13557364; doi:10.1371/journal.pone.0353935)
Supplement: Supplementary file 6 [file pone.0353935.s006.docx]

**S6 Appendix 6.** Results multivariate multinomial logistic regression analysis Part 2 with cut-offs ≥ 4

|  |  |  | Adjusted OR (95% CI) relative to P*hysical aggressive. has conspiracy beliefs* group (Group 4) | | | |  |  |  |  |  |  |  |
| --- | --- | --- | --- | --- | --- | --- | --- | --- | --- | --- | --- | --- | --- |
|  |  | Group 4: *Physical aggressive conspiracy believers* (n=346) | Group 3: *Physical aggressive non-conspiracy believers* (n=420) | | Group 2: *Non-physical aggressive conspiracy believers* (n=243) | |  |  |  |  |  |  |  |
|  | N^total^ |  | n (%) | AOR (95% CI) | n (%) | AOR (95% CI) |  |  |  |  |  |  |  |
| Anxiety and depression symptoms levels (T1a) | | | | | | | | | |  |  |  |  |
| - no (ref.) | 1392 | 191 (43.3 / 47.6) | 250 (56.7) | 1 | 210 (52.4) | 1 |  |  |  |  |  |  |  |
| - moderate | 355 | 96 (45.9 / 82.1) | 113 (54.1) | 0.94 (0.67-1.31) | 21 (17.9) | 0.22 (0.13-0.37)^***^ |  |  |  |  |  |  |  |
| - high | 156 | 59 (50.9 / 83.1) | 57 (49.1) | 0.78 (0.51-1.19) | 12 (16.9) | 0.18 (0.09-0.36)^***^ |  |  |  |  |  |  |  |
| Lack of emotional support (T1a) | | | | | | | | | |  |  |  |  |
| - no (ref.) | 1173 | 139 (38.6 / 45.9) | 221 (61.4) | 1 | 164 (54.1) | 1 |  |  |  |  |  |  |  |
| - medium | 404 | 97 (47.8 / 67.4) | 106 (52.2) | 0.69 (0.48-0.97)^*1^ | 47 (32.6) | 0.45 (0.29-0.69)^***^ |  |  |  |  |  |  |  |
| - high | 325 | 110 (54.2 / 77.5) | 93 (45.8) | 0.54 (0.38-0.76)^***^ | 32 (22.5) | 0.25 (0.16-0.40)^***^ |  |  |  |  |  |  |  |
| Potential traumatic events (in 12 months before T1a) | | | | | | | | | |  |  |  |  |
| - no (ref.) | 1723 | 268 (42.0 / 53.8) | 370 (58.0) | 1 | 230 (46.2) | 1 |  |  |  |  |  |  |  |
| - yes | 180 | 78 (60.9 / 85.7) | 50 (39.1) | 0.45 (0.30-0.67)^***^ | 13 (14.3) | 0.23 (0.12-0.42)^***^ |  |  |  |  |  |  |  |
| Stressful life events (in 12 months before T1a) | | | | | | | | | |  |  |  |  |
| - no (ref.) | 1335 | 209 (42.7 / 54.1) | 280 (57.3) | 1 | 177 (45.9) | 1 |  |  |  |  |  |  |  |
| - yes | 567 | 137 (49.5 / 67.5) | 140 (50.5) | 0.74 (0.55-1.01) | 66 (32.5) | 0.43 (0.29-0.62)^***^ |  |  |  |  |  |  |  |
| Financial problems (T1a) | |  |  |  |  |  |  |  |  |  |  |  |  |
| - no (ref.) | 1783 | 292 (43.0 / 55.8) | 387 (57.0) | 1 | 231 (44.2) | 1 |  |  |  |  |  |  |  |
| - yes | 120 | 54 (62.1 / 81.8) | 33 (37.9) | 0.48 (0.30-0.77)^**^ | 12 (18.2) | 0.26 (0.13-0.51)^***^ |  |  |  |  |  |  |  |
| Life satisfaction (T1b) | | | | | | |  |  |  |  |  |  |  |
| - very high (ref.) | 487 | 42 (34.1 / 38.9) | 81 (65.9) | 1 | 66 (61.1) | 1 |  |  |  |  |  |  |  |
| - high | 271 | 31 (39.7 / 47.7) | 47 (60.3) | 0.78 (0.43-1.41) | 34 (52.3) | 0.74 (0.39-1.40) |  |  |  |  |  |  |  |
| - medium | 405 | 72 (40.2 / 60.5) | 107 (59.8) | 0.79 (0.49-1.28) | 47 (39.5) | 0.41 (0.23-0.70)^**^ |  |  |  |  |  |  |  |
| - low | 332 | 73 (46.2 / 59.8) | 85 (53.8) | 0.63 (0.38-1.03) | 49 (40.2) | 0.45 (0.26-0.78)^**^ |  |  |  |  |  |  |  |
| - very low | 408 | 128 (56.1 / 73.1) | 100 (43.9) | 0.43 (0.27-0.68)^***^ | 47 (26.9) | 0.21 (0.12-0.36)^***^ |  |  |  |  |  |  |  |
| Number assessed problems | | | | | | |  |  |  |  |  |  |  |
| - zero (ref.) | 550 | 43 (35.2 / 36.1) | 79 (64.8) | 1 | 76 (63.9) | 1 |  |  |  |  |  |  |  |
| - one | 530 | 61 (35.9 / 42.1) | 109 (64.1) | 0.98 (0.60-1.60) | 84 (57.9) | 0.69 (0.41-1.15) |  |  |  |  |  |  |  |
| - two | 407 | 90 (47.1 / 66.2) | 101 (52.9) | 0.62 (0.39-1.00)^*1^ | 46 (33.8) | 0.27 (0.16-0.46)^***^ |  |  |  |  |  |  |  |
| - three | 247 | 72 (47.4 / 72.7) | 80 (52.6) | 0.62 (0.37-1.01) | 27 (27.3) | 0.19 (0.10-0.34)^***^ |  |  |  |  |  |  |  |
| - four or more | 171 | 80 (60.6 / 87.9) | 52 (39.4) | 0.35 (0.21-0.60)^***^ | 11 (12.1) | 0.07 (0.03-0.15)^***^ |  |  |  |  |  |  |  |

^1^ Not significant after the Benjamini-Hochberg correction of p-values. ^*^ *p* < 0.05. ^**^ *p* < 0.01. ^***^ *p* < 0.001
