# Supplementary material for: Physically aggressive conspiracy believers often faced various psycho-social problems: Findings from a longitudinal population-based study
Source: PLoS One. 2026 Sep 9;21(9):e0353935. doi: 10.1371/journal.pone.0353935 (PMC13557364; doi:10.1371/journal.pone.0353935)
Supplement: Supplementary file 7 [file pone.0353935.s007.docx]

**S7 Appendix 7.** Number respondents AOR were derived from (besides control variables)

|  | Dependent variables (based on group membership) | | | | | | | | | | | | | | | | | | |
| --- | --- | --- | --- | --- | --- | --- | --- | --- | --- | --- | --- | --- | --- | --- | --- | --- | --- | --- | --- |
|  | group 1-2 | | |  | group 1-3 | | |  | group 1-4 | | |  | group 4-3 | | |  | group 4-2 | | |
|  | Used Cut-offs | | | | | | | | | | | | | | | | | | |
|  | ≥ 6 | ≥ 5 | ≥ 4 |  | ≥ 6 | ≥ 5 | ≥ 4 |  | ≥ 6 | ≥ 5 | ≥ 4 |  | ≥ 6 | ≥ 5 | ≥ 4 |  | ≥ 6 | ≥ 5 | ≥ 4 |
|  | n | n | n |  | n | n | n |  | n | n | n |  | n | n | n |  | n | n | n |
| Anxiety and depression symptoms levels (T1a) | | | | |  |  |  |  |  |  |  |  |  |  |  |  |  |  |  |
| reference - moderate | 1002 | 1044 | 1097 |  | 1029 | 1113 | 1229 |  | 957 | 1040 | 1153 |  | 254 | 421 | 650 |  | 227 | 352 | 518 |
| reference - high | 894 | 937 | 991 |  | 899 | 974 | 1076 |  | 861 | 933 | 1019 |  | 222 | 369 | 557 |  | 217 | 332 | 472 |
| Lack of emotional support (T1a) | | |  |  |  |  |  |  |  |  |  |  |  |  |  |  |  |  |  |
| reference - medium | 926 | 962 | 1014 |  | 951 | 1024 | 1130 |  | 878 | 946 | 1039 |  | 223 | 364 | 563 |  | 198 | 302 | 447 |
| reference - high | 852 | 889 | 935 |  | 887 | 958 | 1053 |  | 837 | 907 | 988 |  | 246 | 387 | 563 |  | 211 | 318 | 445 |
| Potential traumatic events (in 12 months before T1a) | | | | | |  |  |  |  |  |  |  |  |  |  |  |  |  |  |
| reference - yes | 1032 | 1078 | 1137 |  | 1091 | 1186 | 1314 |  | 1022 | 1118 | 1240 |  | 325 | 516 | 766 |  | 266 | 408 | 589 |
| Stressful life events (in 12 months before T1a) | | | | |  |  |  |  |  |  |  |  |  |  |  |  |  |  |  |
| reference - yes | 1031 | 1077 | 1136 |  | 1089 | 1185 | 1313 |  | 1022 | 1117 | 1239 |  | 325 | 516 | 766 |  | 267 | 408 | 589 |
| Financial problems (T1a) | | | | | | | | | | | | | | | | | | | |
| reference - yes | 1033 | 1078 | 1137 |  | 1091 | 1186 | 1314 |  | 1023 | 1118 | 1240 |  | 326 | 516 | 766 |  | 268 | 408 | 589 |
| Life satisfaction (T1b) |  |  |  |  |  |  |  |  |  |  |  |  |  |  |  |  |  |  |  |
| reference - high | 512 | 531 | 557 |  | 501 | 535 | 585 |  | 477 | 498 | 530 |  | 64 | 119 | 201 |  | 75 | 115 | 173 |
| reference - medium | 542 | 563 | 590 |  | 552 | 598 | 665 |  | 514 | 552 | 591 |  | 112 | 196 | 302 |  | 102 | 161 | 227 |
| reference - low | 488 | 508 | 538 |  | 497 | 532 | 589 |  | 462 | 493 | 538 |  | 113 | 179 | 281 |  | 104 | 155 | 230 |
| reference - very low | 498 | 518 | 544 |  | 513 | 557 | 612 |  | 502 | 543 | 601 |  | 153 | 238 | 351 |  | 138 | 199 | 283 |
| Number of assessed problems | |  |  |  |  |  |  |  |  |  |  |  |  |  |  |  |  |  |  |
| reference - one | 726 | 753 | 788 |  | 706 | 751 | 816 |  | 657 | 691 | 732 |  | 107 | 186 | 292 |  | 127 | 188 | 264 |
| reference - two | 586 | 613 | 644 |  | 596 | 639 | 702 |  | 565 | 607 | 655 |  | 117 | 202 | 313 |  | 107 | 176 | 255 |
| reference - three | 476 | 495 | 523 |  | 484 | 525 | 579 |  | 456 | 494 | 535 |  | 100 | 179 | 274 |  | 92 | 149 | 218 |
| reference - four or more | 426 | 445 | 467 |  | 450 | 472 | 511 |  | 443 | 473 | 503 |  | 133 | 185 | 254 |  | 109 | 158 | 210 |
